# Research Interest in Copper Materials for Caries Management: A Bibliometric Analysis

**DOI:** 10.3390/jfb15090274

**Published:** 2024-09-20

**Authors:** Veena Wenqing Xu, Mohammed Zahedul Islam Nizami, Iris Xiaoxue Yin, John Yun Niu, Ollie Yiru Yu, Chun-Hung Chu

**Affiliations:** 1 Faculty of Dentistry, University of Hong Kong, Hong Kong, China; u3008489@connect.hku.hk (V.W.X.); mnizami@forsyth.org (M.Z.I.N.); irisxyin@hku.hk (I.X.Y.); niuyun@hku.hk (J.Y.N.); ollieyu@hku.hk (O.Y.Y.); 2 Department of Mineralized Tissue Biology and Bioengineering, The ADA Forsyth Institute, Cambridge, MA 02142, USA

**Keywords:** caries, antibacterial, prevention, biofilm, copper, nanoparticles

## Abstract

This study examined research interest in copper materials for caries management. We conducted an exhaustive literature search of English publications on copper materials for caries management. We removed duplicate publications and screened the titles and abstracts to identify relevant publications. Then, we analyzed the bibliometric data of the publications using the Bibliometrix and VOSviewer programs. This study included 75 laboratory studies, six clinical trials, and 17 reviews. Most of the original research studied copper or copper oxide nanoparticles (45/81, 56%). The materials could be doped into topical agents, restorative fillers, dental adhesives, dental implants, and orthodontic appliances. Since the first paper was published in 1980, publication counts gradually increased and surged in 2019. Among publications on copper materials for caries management, the publication counts and citations from 2019 to 2024 accounted for 65% (64/98) and 74% (1677/2255) over the last 45 years. Cocitation analysis revealed that the two main keywords were nanoparticles and antibacterial activity, and their burst strengths (period) were 3.84 (2021–2024) and 2.21 (2020–2021). The topics of the top two publications with the highest citation burst strength (period) are the antimicrobial effect of copper oxide nanoparticles (3.14, 2021–2022) and the dental application of copper nanoparticles (2.84, 2022–2024). In conclusion, this study revealed a growing interest in copper materials for caries management.

## 1. Introduction

Dental caries, a chronic and globally pervasive disease, affects approximately 50% of the world’s population, according to a World Health Organization (WHO) survey [1]. Ranking as the fourth most costly ailment to treat, dental caries significantly contribute to the global disease burden and can occur at any stage of an individual’s life. The primary causative factors of this disease include cariogenic microorganisms, the host or tooth surface, substrates, and time [2]. Cariogenic bacteria that colonize tooth surfaces can metabolize fermentable carbohydrates, subsequently producing organic acids. Despite the high mineralization of enamel and dentin, these acids can cause dissolution [3]. The ongoing depletion of minerals leads to the destruction of the tooth structure, ultimately resulting in dental caries [4]. Effective caries management strategies focus on reducing bacterial activity and preventing biofilm formation. In response to this need, researchers have developed various dental materials with antibacterial properties to combat dental caries.

Copper, as a common metal element, has been widely used in various medical applications throughout history. Dating back to 2600–2200 B.C.E., the Smith Papyrus documents the ancient use of copper for treating and preventing ailments [5]. In more recent centuries, records reveal the use of copper preparations to address skin illnesses, syphilis, and tuberculosis [6]. These documents underscore the bactericidal properties of copper with minimal toxicity to human cells.

In recent decades, copper has gained considerable attention for its potential in preventing dental caries. In the 1980s, researchers incorporated copper substances into topical treatments and drinking water to explore its preventive effects against tooth decay [7,8]. The advancement of nanotechnology has facilitated the production of copper-based nanoparticles with antibacterial properties for caries treatment and prevention [9]. These nanoparticles exhibit antimicrobial properties due to their high surface area-to-volume ratio [10]. Furthermore, various copper compounds, including copper salt [11], copper oxide [12], and copper alloy [13], have been formulated for the purpose of caries control. These compounds demonstrate antimicrobial properties while causing minimal damage to human cells [14]. Researchers used copper compounds in dental restorative materials [15], implants [16], and other oral health products [17] to prevent caries. These copper-based materials have been developed to prevent dental caries and have demonstrated promising outcomes.

Bibliometric analysis is a reliable method for assessing research hotspots, tracking publication patterns over time, and identifying influential countries, keywords, and publications in a specific field of study [18]. Various bibliometric research approaches enable researchers to evaluate the research status comprehensively and determine relative trends, guiding future research directions and collaborations in particular areas [19]. Thus, it is both advantageous and essential to investigate the publication status and focal points of this field using a bibliometric analysis approach.

Bibliometric analyses have been extensively employed to investigate the scientific outputs in various fields, including dentistry [20]. Although there is a growing focus on copper materials for caries management [21], no bibliometric investigation has been conducted on this subject. The objective of this study is to investigate and quantify the worldwide research interest in copper materials for caries management. This bibliometric analysis may facilitate in the development of novel copper materials for caries management in future research.

## 2. Methods

### 2.1. Search Strategy

Two independent researchers conducted a comprehensive search of the literature in three common databases PubMed, Scopus, and Web of Science to identify publications on 19 July 2024. The keywords were (copper OR Cu) AND (caries OR tooth decay OR demineralisation OR demineralization OR remineralisation OR remineralising OR remineralization OR remineralizing). The search was restricted to the English literature, without any limitations on the publication date.

### 2.2. Study Selection and Data Extraction

**Figure 1:**
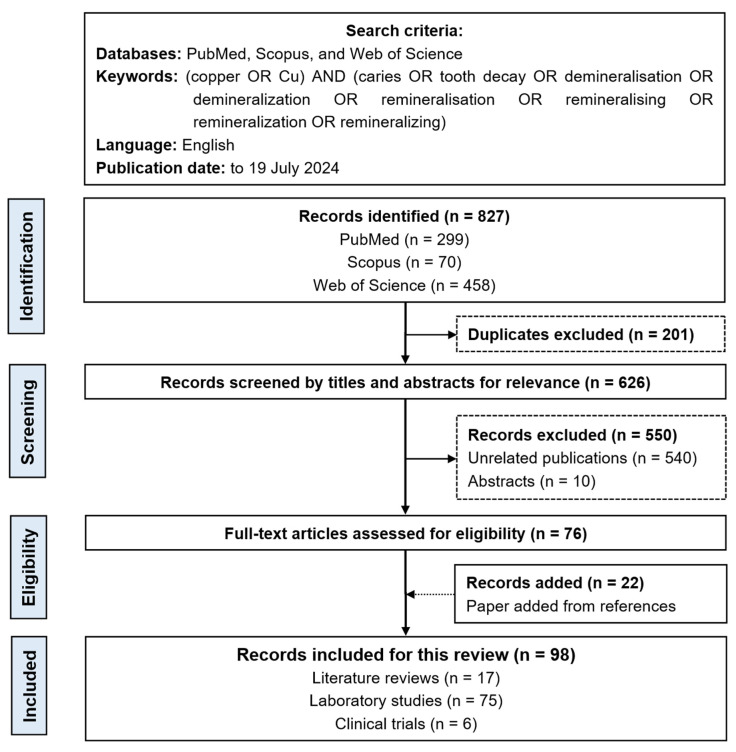
Flow chart of the literature search.

This bibliometric analysis included studies about the copper materials used for caries management. Figure 1 displays the flow chart of the literature search.

Two researchers independently examined and included studies about the copper materials used for caries management. They removed duplicate publications from the three databases. They reviewed the titles and abstracts of the publications to identify potentially eligible publications. They excluded studies not related to copper materials developed for caries management. Subsequently, they retrieved all the texts from the remaining publications for review. After that, the reference lists of the chosen papers were meticulously examined to identify suitable publications. The researchers sought the input of a third investigator to determine the publications included in this bibliometric analysis.

VOSviewer and the R package program Biblioshiny were used to conduct the bibliometric analysis for this study. The researchers used the bar chart and line chart to display the trends in annual publication and citation counts separately. They used another table to present the top 5 journals that have the greatest number of publications and the highest counts of citations. Additional investigations involved examining the cooccurrence and cocitation of references and keywords. The method of burst detection was used to discover publications and keywords that had a sudden and significant rise in frequency over a short period of time, suggesting the current areas of intense investigation. The researchers also used the word cloud to illustrate the keywords in these publications.

## 3. Results

During the preliminary literature search, two researchers discovered 827 papers that may satisfy the eligibility requirements (299 publications in PubMed, 70 publications in Scopus, and 458 publications in Web of Science). They then eliminated 201 duplicate records. After screening the titles and abstracts, 550 publications were removed because they were unrelated publications or abstracts. Twenty-two publications met the inclusion requirements after a search of the references of the chosen publications. Consequently, this study included 98 papers on the use of copper materials for managing dental caries (Figure 1). Most publications (75/98, 77%) were laboratory studies, six publications (6/98, 6%) were clinical research, and seventeen (17/98, 17%) were reviews. The copper materials involved in the 81 original publications (laboratory and clinical studies) were categorized into three groups: copper and copper alloy materials (36/81, 44%), copper salt materials (26/81, 32%), and copper oxide materials (19/81, 23%). Table 1 displays studies of copper materials in caries management based on the three classifications. In addition, most of the copper materials (48/81, 59%) were copper-based nanomaterials, including copper nanoparticles (n = 29), copper oxide nanoparticles (n = 16), and copper iodide nanoparticles (n = 3).

**Figure 2:**
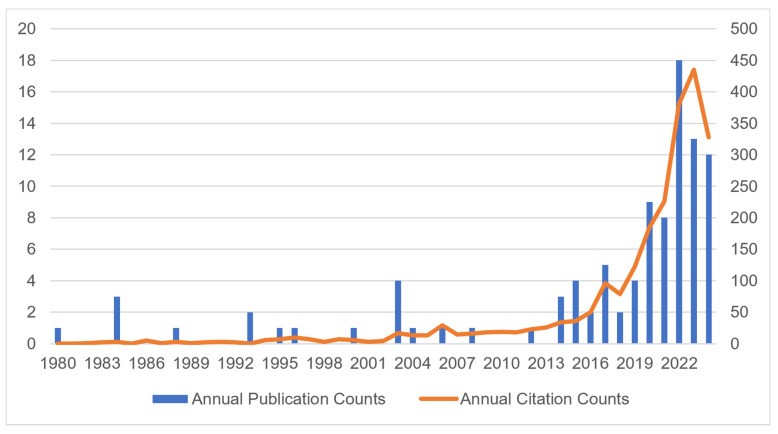
Annual publications and citation count on copper materials for caries management from 1980 to 2024.

Figure 2 depicts the patterns in yearly publication and citation counts. The initial publishing occurred in 1980. Since 2008, there has been a gradual increase in both the annual publication counts and citation counts, which experienced a significant jump starting in 2019. Among publications on copper materials for caries management, 65% (64/98) of the total publications were published in the past five years. Figure 2 illustrates three distinct peaks in the frequency of publications: five publications in 2017 and nine publications in 2020, followed by a peak of eighteen in 2022. There were 1677 citations from 2019 to 2024 of the included publications, which represents 74% of the total number of citations (2255) throughout the past 45 years. In addition, the number of publications released each year has significantly increased.

Table 2 provides a summary of the top five journals that publish research on copper materials for caries management, based on the publication number and total citations, respectively. The journals that published the most research on copper were *Caries Research* (n = 6), *Dental Materials* (n = 5), *Journal of Dentistry* (n = 4), *Nanomaterials* (n = 3), and *Journal of Dental Research* (n = 3). The ranking of the total citations of the journal was different from the publication number of the journals. The journal *Scientific Reports* had the highest citation count (210), with only two publications. It was followed by *Dental Material* (150, with five publications), *Caries Research* (149, with six publications), *Chemical Engineering Journal* (134, with two publications), and *Progress in Natural Science: Materials International* (134, with one publication).

**Figure 3:**
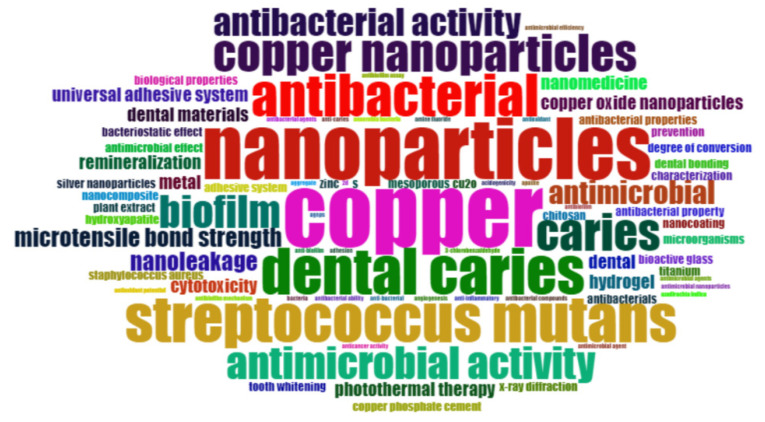
Word cloud of keywords of the publications on copper materials for caries management.

Figure 3 displays the word cloud representing the keywords found in the included publications. Considering all the publications, the prominent keywords were ‘copper’, ‘nanoparticles’, ‘dental caries’, ‘*Streptococcus mutans*’, and ‘antibacterial’.

**Figure 4:**
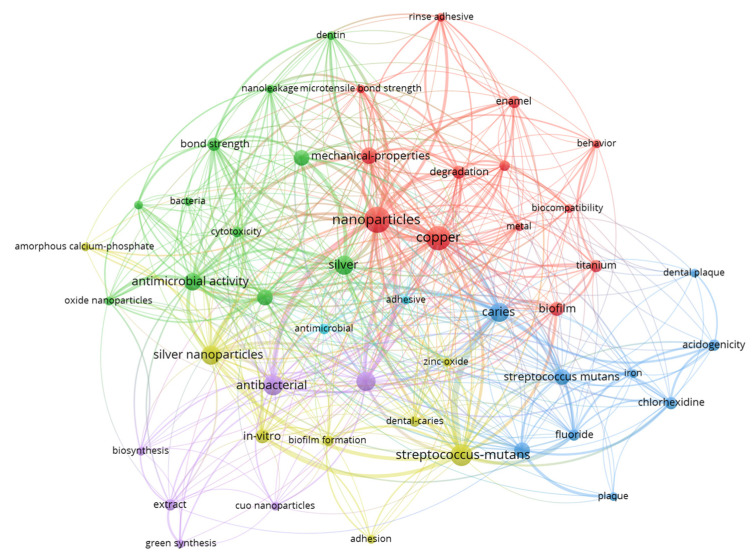
Connections between publications on copper materials for caries management.

Figure 4 illustrates the connections between publications on copper materials for caries management. The keywords are represented by nodes, with the size of each node indicating the frequency of occurrence of the respective keyword. The thickness of the lines between the nodes signifies the strength of their relationships. Closely associated keywords form clusters, which are represented by nodes of the same color. The red and green clusters highlight the characteristics of copper materials for caries management, including keywords such as ‘mechanical properties’ and ‘bond strength’. The purple, yellow, and blue clusters primarily encompass keywords related to the antibacterial effects of copper materials, as indicated by keywords like ‘antibacterial’ and ‘*Streptococcus mutans*’.

Table 3 displays the top 10 keywords in the past decade based on the burst strength. The keyword ‘nanoparticles’ exhibits the highest burst strength value of 3.84 during the burst period from 2021 to 2024. The highest burst strength of ‘nanoparticles’ suggests that copper material for caries management is mostly centered around nanotechnology. The keywords ‘antibacterial activity’ and ‘mechanical property’ have the following highest burst strength (period) of 2.21 (2020–2021) and 1.92 (2019–2024) in the past decade, respectively.

**Figure 5:**
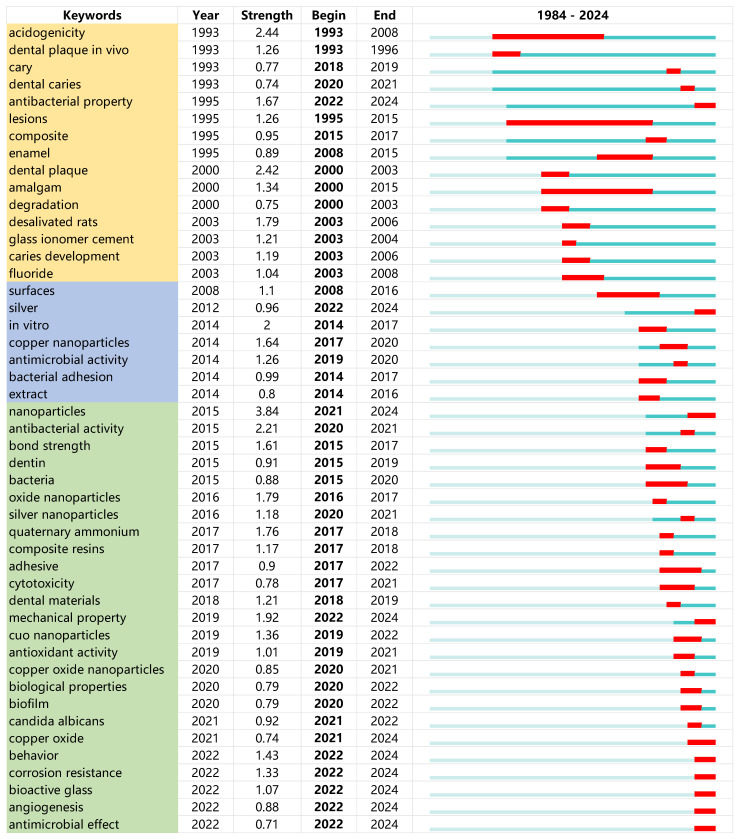
Burst keywords according to the years in these publications.

Figure 5 demonstrates the keywords ranked by burst year provided a dynamic perspective of research interest at different times. Before 2004, copper materials were extensively studied for their antibacterial properties and in vivo effectiveness in managing caries. This was proved by research focusing on terms such as ‘acidogenicity’, ‘antibacterial property’, ‘dental plaque in vivo’, and ‘desalivated rats’. During the subsequent decade, there was a sustained emphasis on antibacterial characteristics, as seen by the proliferation of keywords such as ‘antimicrobial activity’ and ‘bacterial adhesion’. In addition, researchers began to closely monitor copper nanoparticles, as indicated by the keyword ‘copper nanoparticles’. Between 2004 and 2014, there was a noticeable change from in vivo studies to in vitro studies in the publications, as shown by the keyword ‘in vitro’. Over the past decade, there has been a surge in the use of nanotechnology for managing caries in copper materials. This involves the utilization of keywords such as ‘nanoparticles’, ‘oxide nanoparticles’, ‘cuo nanoparticles’, and ‘copper oxide nanoparticles’. Furthermore, the researchers included copper materials into varied matrices and examined the mechanical properties of the materials, as indicated by the keywords ‘adhesive’, ‘composite resins’, ‘mechanical property’, and ‘corrosion resistance’.

Table 4 presents the top 10 references with the highest citation burst strength. The publication with the highest burst strength is an in vitro laboratory research investigating the antimicrobial effect of copper oxide nanoparticles against oral pathogens. The study probed the potent antimicrobial properties of copper nanoparticles against *Streptococcus mutans*, *Lactobacillus acidophilus*, *Lacticaseibacillus casei*, *Candida albicans*, *Candida krusei*, and *Candida glabrata* [22]. The authors suggested that the copper oxide nanoparticles might be employed as a potential control agent to prevent tooth caries and other dental infections. A narrative review holds the second-highest citation burst strength among the references. This review proposed the bactericidal mechanism of copper nanoparticles and discussed the application of copper nanoparticles in dentistry [23]. The third most highly cited reference is an in vitro laboratory study that examines the impact of incorporating copper nanoparticles at different concentrations into an etch-and-rinse adhesive on antimicrobial activity, mechanical properties, and the durability of resin–dentine interfaces. The researchers stated that the addition of 0.5 wt.% copper nanoparticles into adhesives may provide antimicrobial properties and prevent the degradation of the adhesive interface while maintaining the mechanical qualities of the formulations [24].

**Figure 6:**
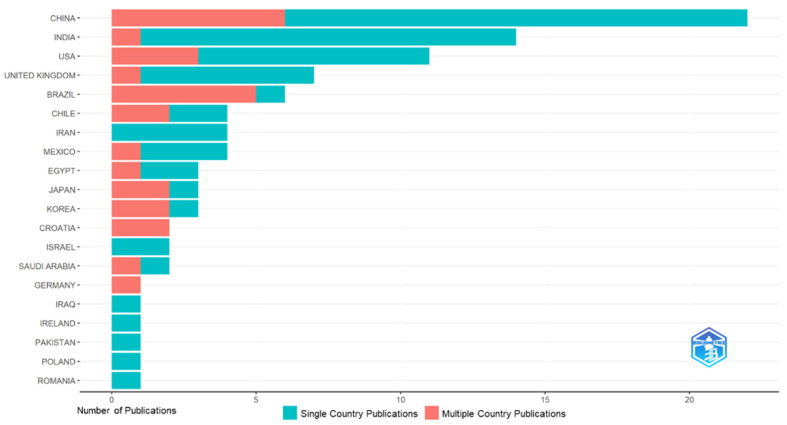
Number of publications on copper materials for caries management according to country.

Figure 6 displays the number of publications on copper materials for caries management according to country. Out of the total 98 publications, China (*n* = 22), India (*n* = 14), and the USA (*n* = 11) collectively accounted for nearly half of the publications (47/98, 48%). Brazil frequently engaged in international collaborations in copper materials for caries management, with a ratio of five collaborations of the MCP (Multiple Country Publications) to one SCP (Single Country Publication). China (MCP/SCP = 6/16) and the US (MCP/SCP = 3/8) were also engaged in international collaborations.

## 4. Discussion

Copper materials possess antibacterial properties against cariogenic bacteria but maintain low toxicity towards human cells. Copper compounds are easily affordable and accessible for production. Hence, copper materials have the potential to serve as replacements for conventional metal antibacterial agents such as silver. A growing number of researchers are interested in studying copper materials for the purpose of managing caries [32]. However, due to the diverse range of copper materials that have been studied, it is challenging to comprehend and categorize previous findings in this sector and initiate new studies [21]. While there is an increasing emphasis on the use of copper materials for managing caries [33], no bibliometric study has been carried out on this topic. This study is the first bibliometric analysis of copper materials, aiming to examine and measure the research interest in copper materials for caries management. This bibliometric analysis provides a comprehensive summary of the latest advancements of research in copper materials for caries management and is a valuable tool for researchers to identify the research hotspots, references, and journals on this topic. This outline can facilitate the advancement of innovative copper materials for caries management in future studies.

Bibliometric analysis is applicable to all fields of study due to its capacity to help researchers understand prior discoveries and stimulate the development of novel concepts [34]. Nevertheless, the necessity of complex stages, numerous analyses, and mapping software tools make bibliometric research difficult [35]. To confront these obstacles, comprehensive open-source tools, including Bibliometrix and VOSviewer, were undertaken to conduct bibliometric analysis [36]. In this study, we employed three common databases in the field of medicine and dentistry, Scopus, PubMed, and Web of Science, to identify publications for inclusion [37]. These databases provided thorough citation information for the included publications. Bibliometrix and VOSviewer could be utilized to conduct a bibliometric analysis based on these thorough and dependable bibliometric data [38].

Bibliometric analysis primarily focuses on quantitative metrics like the number of publications and citation counts. It generates quantitative metrics and offers insights into the research impact. However, it does not assess the research quality. Bibliometric analysis uses citation counts to gauge the impact of a publication, but the number of citations does not necessarily correlate with the quality of the publications.

A total of 163 papers were considered for analysis in this study. The inaugural publication took place in 1980, signifying that the researcher first noted the copper materials for caries management. There has been a steady rise in both the number of publications and the number of citations per year from 2008 onwards and a notable surge in 2019. Over the last five years, citations and publications have accounted for more than 70% of the total number of citations and over 60% of the total number of publications throughout the past 45 years. The increase in the number of publications and citations indicates that academics are closely focusing on copper materials in the field of caries management.

The five journals with the highest number of publications are *Caries Research*, *Dental Materials*, *Journal of Dentistry*, *Nanomaterials*, and *Journal of Dental Research*. The five journals with the highest number of citations are *Scientific Reports*, *Dental Materials*, *Caries Research*, *Chemical Engineering Journal*, and *Progress in Natural Science: Materials International*. The journal *Scientific Reports* stands out for having the highest citation total while having only two publications. The reason for this is a laboratory study conducted in vitro, which provided 201 citations. This laboratory investigation showed that the copper-bearing titanium alloy exhibits antibacterial properties against *Streptococcus mutans* and *Porphyromonas gingivalis*, suggesting its potential for dental implant and caries management [13]. Furthermore, the journal *Chemical Engineering Journal* achieved the fourth-highest citation count, despite only having two publications. This impressive citation count was primarily attributed to a review, which garnered a remarkable 112 citations. This review shares a topic similar to the above study on copper-bearing titanium alloy. It specifically discusses copper-doped titanium nanocomposite antimicrobial coatings for dental implants [39]. In addition, the journal *Progress in Natural Science-Materials International* achieved the fifth-highest citation count despite having only one publication. This in vitro laboratory investigation successfully synthesized copper and bimetallic copper–nickel nanoparticles and confirmed their antibacterial properties against *Streptococcus mutans*, *Staphylococcus aureus*, and *Escherichia coli*. This publication received an impressive 134 citations [40].

Keyword burst detection is a valuable analytical technique used to identify the keywords that experience a sudden surge in the research of copper materials in caries management. The investigation into the use of copper materials for caries management began with animal studies, specifically focusing on the burst keywords ‘dental plaque in vivo’ and ‘desalivated rats’ before 2004. In 1984, Afseth et al. revealed that the application of copper sulfate resulted in a significant reduction in caries scores in rats [41]. The researchers also assessed the impact of combining topically applied copper with fluoride in the drinking water. They found that the groups of animals receiving a combination of copper and fluoride had significantly fewer instances of tooth decay compared to the groups given either substance alone at the same concentrations [8]. However, the publications exhibited a discernible shift from in vivo investigations to in vitro studies, as seen using the keyword ‘in vitro’ starting from 2004. The change may be attributed to the identification of cariogenic microorganisms [42]. These in vitro studies investigated the antibacterial effects of novel copper materials in vitro. In addition, people have turned their attention to natural extracts, which has emerged as another prominent trend in 2014. Natural extracts often exhibit great biocompatibility, causing fewer adverse reactions compared to synthetic materials [43]. Additionally, the natural extracts are derived from renewable resources, making them environmentally friendly [44]. A study utilized a simple and environmentally friendly biological approach to produce copper oxide nanoparticles by using an extract from the Nilgirianthus ciliatus plant. The researchers confirmed that the nanoparticles demonstrate antibacterial activities against *Streptococcus mutans* [45]. In addition, researchers initiated close surveillance of copper nanoparticles starting in 2014, as evidenced by the keyword ‘copper nanoparticles’. Researchers have been focusing more on copper materials at the nanoscale, probably because the nanoparticles exhibit excellent antibacterial effects [46]. Between 2014 and 2024, the terms ‘nanoparticles’, ‘oxide nanoparticles’, ‘cuo nanoparticles’, and ‘copper oxide nanoparticles’ experienced a significant increase in popularity. Researchers synthesized various copper-based nanomaterials and employed them to prevent tooth decay [47,48]. Furthermore, the researchers doped copper components into several matrices and analyzed the mechanical characteristics of the materials, as indicated by the keywords ‘adhesive’, ‘composite resins’, ‘mechanical property’, and ‘corrosion resistance’. For example, a study incorporated polyacrylic acid-coated copper iodide nanoparticles into dental adhesives and evaluated the antibacterial properties and bond strength in the adhesives [49]. Future research direction may include developing copper natural extracts with nanoscale for caries management.

Out of the top 10 references with the highest citation burst strength in the last decade, seven were laboratory research and three were reviews. Of the ten publications, seven publications specifically studied nanoparticles made of copper. Out of these, five publications focused on copper nanoparticles, while the other two studies focused on copper oxide nanoparticles. Furthermore, seven studies analyzed the antibacterial efficacy of copper materials, while four publications explored the mechanical characteristics of copper materials incorporated into different dental materials. The hotspots of the top ten references with the highest citation burst strength coincide with those identified by the keyword burst.

The majority of the articles included in the study utilized *Streptococcus mutans* as the research subjects to investigate the antibacterial properties of copper materials. It is important to mention that cariogenic species should not be restricted solely to *Streptococcus mutans* [50]. In addition to Streptococcus mutans, other microorganisms such as *Streptococcus sobrinus* and *Candida albicans* may play a role in the development of dental caries [51,52]. Moreover, it is crucial to note that the primary emphasis of research has been on in vivo animal studies and in vitro laboratory investigations. It is important to mention that just six clinical trials, comprising just 6% of the total, were included in this research. In vitro studies and animal studies cannot fully replicate the physiological conditions of the human body, such as immune response and interactions with other tissues and organs [53]. Therefore, the findings from in vitro studies may not directly translate to clinical outcomes. Furthermore, in vitro studies do not provide information on safety, absorbency, and excretory in humans [54]. Clinical trials are essential for determining the appropriate dosage, potential side effects, and overall safety profile. In summary, while in vitro studies are crucial for the initial exploration of copper materials for caries management, further clinical trials are indispensable for confirming the safety and efficacy of the copper materials.

We compared this bibliometric analysis to other bibliometric analyses focusing on materials for caries management. Our analysis identified an increase in publications on copper materials for caries management over the past five years, which aligns with the trends observed in studies on fluoride varnish [55] and antimicrobial peptides [56] used for caries management. However, the growth rate in publications on copper materials appears to be more pronounced compared to these other materials, suggesting a burgeoning interest and potential advancements in the use of copper materials for caries management. Additionally, similar to the findings in the bibliometric analysis of fluoride varnish in dentistry [55], our study found that journals such as Caries Research and Journal of Dental Research are leading publication venues for research on novel materials for caries management. In terms of keywords, the prevalent research themes in our analysis, such as antibacterial, antimicrobial, and bond strength, were also commonly observed in the bibliometric analyses of silver diamine fluoride in dentistry [57]. For example, a study highlighted the focus on the antimicrobial effect of materials on caries management, which is also central to our findings [58]. The results suggest that our bibliometric analysis not only aligns with but also expands upon existing research trends in antimicrobial agents within dentistry.

As a bibliometric analysis, this study may have potential biases in citation practices. The authors may cite their own work frequently to increase their citation counts. In addition, articles published in high-impact journals and in English are more likely to be cited [59].

This study utilized Scopus, Web of Science, and PubMed as the sources for this bibliometric analysis. These three databases offer a robust and comprehensive foundation due to their extensive coverage of peer-reviewed literature [60]. However, some research might be excluded if it is published in journals that are not indexed by these databases. Additionally, these databases predominantly indexed peer-reviewed journal articles, which means important insights from books or reports from organizations might be overlooked [37]. This could lead to an incomplete picture of the available literature. Furthermore, this study limited the search to English publications. While English is often considered the lingua franca of academia and a significant proportion of high-impact research is published in English-language journals, this approach may introduce potential biases. Research conducted in non-English-speaking countries might be missed, leading to a dominance of perspectives and findings from English-speaking countries [61]. Different regions and cultures may have unique approaches, findings, and interpretations of research. Limiting the search to English publications may result in a lack of diversity in perspectives and methodologies, potentially overlooking important studies [62]. Future studies may consider including studies that are not published in English to provide a comprehensive and balanced understanding of the copper materials in caries management.

## 5. Conclusions

Since the first study on copper materials for caries management was published in 1980, researchers have been actively investigating this topic. Notably, two-thirds of the publications have been released in the last five years, starting from 2018. Most publications consisted of laboratory investigations that examined the mechanical and antibacterial properties of copper nanoparticles.

## Figures and Tables

**Table 1:** Studies of copper materials in caries management.

Category	Material	Property	Application
Copper and copper alloy materials	Cu nanoparticles, Cu-Ni nanoparticles, Cu-Ti alloy, Cu-Fe alloy	Inhibit the growth of *S. mutans*, *S. aureus*, *S. sanguinis*, *E. coli*, *P. gingivalis*, and *S. sanguinis*; Improve the substrate’s mechanical properties such as micro-tensile bond strength, nanoleakage, microhardness, ultimate tensile strength, durability, cross-linking density, solubility, and corrosive effects.	Topical agent, Dental adhesive, Restorative filler, Dental implant, Orthodontic appliances
Copper salt materials	CuSO_4_ solution, Cu_3_(PO_4_)_2_ solution, CuF_2_ solution, CuCl_2_ solution, CuI nanoparticles	Inhibit the growth of *S. mutans*, *E. faecalis*, *S. sobrinus*, *L. acidophilus*, and *C. albicans*; Improve mechanical properties such as ultimate tensile strength and microhardness.	Topical agent, Dental adhesive, Restorative filler, Mouthwash, Drinking water, Sugar
Copper oxide materials	CuO cement, CuO nanoparticles	Inhibit the growth of *S. mutans*, *L. acidophilus*, *L. casei*, *C. albicans*, *C. krusei*, *C. glabrata*, *S. aureus*, *E.coli*, *S. sobrinus*, *P. aeruginosa*, and *S. salivarius*; Improve the substrate’s mechanical properties such as micro-tensile bond strength, slow and sustained release profile, water sorption, and solubility.	Topical agent, Dental adhesive, Restorative filler, Orthodontic appliances

**Table 2:** Top five journals reporting copper materials for caries management according to the number of publications and total citations.

Journal	Publication Number	Total Citation
Top five journals with highest publication number		
*Caries Research*	6	149
*Dental Materials*	5	150
*Journal of Dentistry*	4	96
*Nanomaterials*	4	59
*Journal of Dental Research*	3	60
Top five journals with highest citation counts		
*Scientific Reports*	2	210
*Dental Materials*	5	150
*Caries Research*	6	149
*Chemical Engineering Journal*	2	134
*Progress in Natural Science: Materials International*	1	134

**Table 3:** Top 10 keywords with highest burst strength in the past ten years.

Keywords	Strength	Burst Period
nanoparticles	3.84	2021–2024
antibacterial activity	2.21	2020–2021
mechanical property	1.92	2022–2024
oxide nanoparticles	1.79	2016–2017
quaternary ammonium	1.76	2017–2018
antibacterial property	1.70	2020–2024
copper nanoparticles	1.64	2017–2020
bond strength	1.61	2015–2017
behavior	1.43	2022–2024
cuo nanoparticles	1.36	2019–2022

**Table 4:** Top 10 references with highest citation burst strength in the past ten years.

Reference (Title, Year, Journal, Citation)	Strength	Burst Period
Antimicrobial Effect of Copper Oxide Nanoparticles on Some Oral Bacteria and Candida Species, 2017, *Journal of Dental Biomaterials* [22]	3.14	2021–2022
Application of Copper Nanoparticles in Dentistry, 2022, *Nanomaterials* [23]	2.84	2022–2024
The role of copper nanoparticles in an etch-and-rinse adhesive on antimicrobial activity, mechanical properties and the durability of resin-dentine interfaces, 2017, *Journal of Dentistry* [24]	2.51	2020–2022
Biological, mechanical and adhesive properties of universal adhesives containing zinc and copper nanoparticles, 2019, *Journal of Dentistry* [25]	2.34	2022–2024
The Effect of CuO Nanoparticles on Antimicrobial Effects and Shear Bond Strength of Orthodontic Adhesives, 2018, *Journal of Dentistry* [26]	2.30	2020–2021
An evaluation of the antibacterial properties and shear bond strength of copper nanoparticles as a nanofiller in orthodontic adhesive, 2015, *Australian Orthodontic Journal* [27]	2.22	2017–2020
A systematic review about antibacterial monomers used in dental adhesive systems: Current status and further prospects, 2015, *Dental Materials* [28]	2.02	2017–2019
All-ceramic or metal-ceramic tooth-supported fixed dental prostheses (FDPs)? A systematic review of the survival and complication rates, 2017, *Dental Materials* [29]	1.69	2021–2022
Resin-based composite performance: are there some things we can’t predict, 2013, *Dental Materials* [30]	1.64	2017–2018
Antibacterial activity of a glass ionomer cement doped with copper nanoparticles, 2020, *Dental Materials Journal* [31]	1.61	2022–2024

## Data Availability

Not applicable.
